# No Regard for Those Who Need It: The Moderating Role of Follower Self-Esteem in the Relationship Between Leader Psychopathy and Leader Self-Serving Behavior

**DOI:** 10.3389/fpsyg.2018.01281

**Published:** 2018-07-24

**Authors:** Dick P. H. Barelds, Barbara Wisse, Stacey Sanders, L. Maxim Laurijssen

**Affiliations:** ^1^Department of Organizational Psychology, University of Groningen, Groningen, Netherlands; ^2^Durham University Business School, Durham University, Durham, United Kingdom; ^3^Department of Economics and Business, University of Groningen, Groningen, Netherlands

**Keywords:** self-serving behavior, psychopathy, self-esteem, leadership, followership, Dark Triad

## Abstract

Recent instances of corporate misconduct and examples of blatant leader self-serving behavior have rekindled interest in leader personality traits as antecedents of negative leader behavior. The current research builds upon that work, and examines the relationship between leader psychopathy and leader self-serving behavior. Moreover, we investigate whether follower self-esteem affects the occurrence of self-serving behavior in leaders with psychopathic tendencies. We predict that self-serving behaviors by psychopathic leaders are more likely to occur in the interaction with followers low in self-esteem. We first conducted an experimental study (*N* = 156), in which we manipulated follower self-esteem, measured leader psychopathy, and assessed their combined effect on leader self-serving behavior using an ultimatum game. We then conducted a multi-source field study (*N* = 124 leader–follower dyads) using questionnaires to assess leader psychopathy, follower self-esteem, and perceived leader self-serving behavior. Across both studies, we found that leader psychopathy was positively related to their self-serving behavior, but only when followers had low rather than high self-esteem. As expected, our studies showed that the degree to which (perceived) psychopathic traits of leaders are reflected in their behavior depends on the characteristics of their followers. Apparently, the behavioral expression of negative leader traits is not only a matter of the trait strength, but instead is the result of the interplay between leader and follower in a certain context.

## Introduction

Although leaders are expected to take group and subordinate interests in consideration when making decisions (Northouse, 2004), some leaders clearly fail to do so. Indeed, recent media accounts have made blatantly clear that some leaders act self-servingly and disregard the needs of others. A recent example is Martin Shkreli who hiked up the price of popular AIDS medicine Daraprim by 5,000% – from $13.50 to $750 – and who was arrested after being accused of running a “Ponzi scheme” in order to pay for personal debts. Not only does such self-serving behavior lead to angry and shocked responses from the general public, research has pointed out that it also undermines the effectiveness and functioning of organizations and the people working in them. Indeed, compared to group or employee focused leaders, self-serving leaders contribute negatively to organizational performance and employee functioning (e.g., Carmeli and Sheaffer, 2009; Mayer et al., 2009; Peterson et al., 2012; Kalshoven et al., 2013; Williams, 2014). In order to mitigate the apparent surplus of self-serving behavior in some leaders, it may be helpful to understand how we can account for it.

In this article, we put forward the hypothesis that leader psychopathy is positively related to their self-serving behavior and disregard for other peoples’ interest. Recent research has shown that psychopathic traits (the tendency to be manipulative, callous, egocentric, and a lack of empathy) may explain a fair amount of destructive leadership, unethical behavior, conflict, immoral decision making, and other types of corporate misconduct (cf. Boddy, 2011; Wu and LeBreton, 2011; Smith and Lilienfeld, 2013; Spain et al., 2014). Given the self-interested and uncaring features of the trait, we expect leader psychopathy to also be related to the extent to which leaders use their position to satisfy their self-serving needs at the expense of their followers.

Importantly, however, Trait Activation Theory stipulates that while a trait is unlikely to change, the nature and frequency of its expression can be altered by the context in which people operate (Christiansen and Tett, 2008; also see Padilla et al., 2007; Krasikova et al., 2013; McCabe and Fleeson, 2016). Logically, the same should hold for leader psychopathy. Because leaders always operate in a context where followers are present, we investigate whether follower characteristics affect the occurrence of self-serving behavior in leaders with psychopathic tendencies. We will focus on follower self-esteem, or follower overall self-evaluation, because it has been found to greatly affect their functioning in organizations (Bowling et al., 2010; Kuster et al., 2013). Those with low self-esteem are likely to be compliant and more susceptible to the ill-treatment of others (cf., Bowling and Beehr, 2006). Moreover, psychopaths seem to have a certain prowess in picking up on the vulnerability of others (Wheeler et al., 2009). We expect, therefore, leader psychopathy to be more strongly positively related to leader self-serving behavior when followers have low self-esteem, and that follower high self-esteem can act as a buffer against self-serving tendencies of leaders with psychopathic traits.

All in all, this research aims to increase our understanding of when leaders are more likely to make self-serving decisions. We point to the interplay of notable leader and follower individual difference variables as an important precursor for such behaviors. In doing so, we aim to highlight that the social context in which a leader operates may affect the likelihood that leader traits will be reflected in their self-serving behavior. Finally, our study may provide more insight into factors that could prevent employees from becoming the victims of those willing to exploit others, and help those who are already targeted to cope more effectively with the self-serving behaviors of their leaders.

### Psychopathy in Leaders and Self-Serving Decision-Making

Psychopathy is part of the *Dark Triad*, a constellation of three personality traits: Machiavellianism, psychopathy, and narcissism (Paulhus and Williams, 2002). All three refer to short-term, self-serving, exploitive social strategies that are positively associated with disagreeableness, and the use of dishonest and manipulative behaviors (e.g., Jonason and Webster, 2010). Psychopathy, however, is often considered to be the darkest trait of these three, because it is unique in its coldness (Paulhus, 2014). Moreover, whereas all three Dark Triad traits are characterized by selfishness, those high in psychopathy are also characterized by recklessness (Jones, 2014; Jones and Paulhus, 2017), and the display of antisocial behaviors. In addition, individuals high in psychopathy do not respond well to punishment (e.g., Jones, 2014). As a result, they are likely to harm others for their own gain (Jones, 2014), even in high-risk situations (e.g., Jones and Paulhus, 2017). In line with this, Laurijssen and Sanders (2016) reported those high in psychopathy to have a hostile attitude toward coworkers, and to be characterized by greed. Given the fact that psychopathy appears to be the Dark Triad trait that is most likely to be related to self-serving behavior, the focus of the present study will therefore be on psychopathy, instead of all three Dark triad traits (cf. Jones, 2014).

The Dual Process model (Levenson et al., 1995; Fowles and Dindo, 2009) distinguishes between two forms of psychopathy: primary “emotionally stable” psychopathy, and secondary “reactive” psychopathy. Individuals with higher levels of primary psychopathy are characterized by their manipulativeness, glibness, egocentricity, callousness, and a general lack of empathy and guilt, whereas those with higher levels of secondary psychopathy display impulsive behavior, a need for stimulation, a parasitic lifestyle, and anti-social felonious tendencies. Notably, whereas individuals who score high on both primary and secondary psychopathy often end up incarcerated, the ones that only score high on primary psychopathy fare relatively well in society (Fowles and Dindo, 2009). Probably as a result of this, and the fact that primary psychopathy seems to capture most of the core of the psychopathy concept (cf. Lykken, 1995; Murphy and Vess, 2003), studies in organizational psychology, including the present one, focus on primary psychopathy.

Employees scoring high on primary psychopathy often obtain relatively high ranked positions in organizations (e.g., Babiak and Hare, 2006; Lilienfeld et al., 2012; Howe et al., 2014; Brooks and Fritzon, 2016). It has been argued that the so-called *successful psychopath*, or the *corporate psychopath* owns his/her success to the employment of effective communication styles, strategic thinking, impression management skills, and charisma (e.g., Babiak and Hare, 2006; Babiak et al., 2010; Harms et al., 2011; Smith and Lilienfeld, 2013). So, although psychopathic traits have some beneficial effects in the work context (beneficial for the person with the psychopathic traits), they are also known to have some damaging ones (damaging for the organization and its employees).

Specifically, psychopathic leaders seem to have lower objective performance levels (Babiak et al., 2010; O’Boyle et al., 2012), and are more likely to engage in risky and/or unethical decision-making (Stevens et al., 2012; Jones, 2014). Moreover, psychopathy has been positively related to counterproductive work behavior (CWB) (O’Boyle et al., 2012), white-collar crime (Ragatz et al., 2012), corporate misbehavior (Clarke, 2005), bullying, and abusive supervision (Boddy, 2011; Laurijssen et al., 2016, unpublished). In addition, leader psychopathy has been negatively related to individual consideration (Westerlaken and Woods, 2013), and employee well-being and satisfaction (Mathieu et al., 2014).

So far, there has not been any notable research attention to the relationship between psychopathy and self-serving leader behavior. Leader self-serving behavior reflects both acts aimed at securing higher monetary benefits for oneself, as well as making self-serving causal attributions, such as taking unwarranted credit for a group accomplishment or by denying responsibility for failure when it comes to group projects (cf. Weary Bradley, 1978; Rus et al., 2010). It may therefore be distinguished from detrimental leadership behaviors such as abusive supervision (cf. Tepper, 2000), or common types of CWBs (cf. Robinson and Bennett, 1995; Spector et al., 2006). Abusive supervision may be defined as “subordinates’ perceptions of the extent to which their supervisors engage in the sustained display of hostile verbal and non-verbal behaviors, excluding physical contact” (Tepper, 2000, p. 178). CWB on the other hand is an umbrella term that may be defined as “any intentional behavior on the part of an organization member viewed by the organization as contrary to its legitimate interests” (Sackett and DeVore, 2001, p. 145). As such, CWB has been proposed to be at the top of a hierarchy, with lower level group factors such as organizational and interpersonal CWB (e.g., Bennett and Robinson, 2000), and more specific behaviors (such as theft, drug or alcohol use, poor attendance, etc.) below these group factors (Sackett and DeVore, 2001). Although measures for these concepts might include one or two items referring to self-interested behaviors (e.g., Schmid et al., 2017), these concepts do not explicitly include self-serving behavior in their definitions.

Given what we know about psychopathy, we can expect a positive relationship between psychopathy and leader self-serving behavior. Those with psychopathic traits are considered to be egotistic and manipulative (Jonason and Webster, 2010), and it has been argued that psychopathic traits may facilitate the effective and unremorseful exploitation of others for personal gain due to a lack of empathic concern (Jonason and Krause, 2013; Jonason et al., 2013). Leaders with psychopathic traits may likewise engage in self-serving behavior at the costs of others, especially because the leader role often comes with power, and power increases the likelihood that people will behave according to their traits (cf., Williams, 2014). Moreover, those scoring high on psychopathy often perceive their workplace as competitive (Jonason et al., 2015), arguably as a function of their competitive orientation (Ten Brinke et al., 2015), which may further enhance the likelihood that resources will be claimed for personal benefit at the expense of others. Therefore, we expect that:

(1) *Hypothesis 1*: Leader psychopathy will be positively related to leader self-serving behavior.

Yet, the extent to which negative leader traits are manifested in their behavior is not only a matter of the strength of the trait (cf. Padilla et al., 2007; Krasikova et al., 2013). Leaders do not operate in a vacuum, instead the leadership role is highly social in nature and followers are part and parcel of the leadership process (Uhl-Bien et al., 2014). To understand and predict leader behavior one needs to consider the leader as well has her or his followers in their particular context and take their interaction into account (Padilla et al., 2007). In this paper, we focus on one characteristic of followers that has been coined as potentially important when studying the consequences of leader psychopathic traits: self-esteem (Thoroughgood et al., 2012).

It has been argued that some people are chronically more likely to fall victim to all sorts of negative interpersonal behaviors, including being the ones that receive the short of the end of the stick when it comes to the division of resources (Zapf and Einarsen, 2003). Knowledge about what characterizes target followers of the harmful behaviors of leaders scoring high on psychopathy can help identify individuals who may be in need of help, now or in the future. Moreover, it could be used to develop interventions aimed at (1) preventing vulnerable individuals becoming the victims of those eager to exploit others, and (2) helping those who are already targeted to cope more adequately with the self-serving behaviors of their leaders.

### The Role of Follower Self-Esteem

Self-esteem is a personal evaluation reflecting what people think of themselves as individuals. As such, it refers to an individual’s overall self-evaluation of his/her competencies (Rosenberg, 1965). Self-esteem also has an affective component: people with high self-esteem like who and what they are, and people with low self-esteem do not (Pelham and Swann, 1989). People with low self-esteem are often attracted to others they believe can provide them direction, the possibility to be a more ‘desirable’ or ‘better’ person, an increased sense of self-worth, and/or a sense of belonging (Howell and Shamir, 2005; Padilla et al., 2007; Thoroughgood et al., 2012). As such, they often turn to their leaders and develop a strong desire to emulate and garner approval from that leader (Howell and Shamir, 2005). Their desire to gain acceptance and approval from their leader also explains their motivation to be compliant and their susceptibility to exploitation (Barbuto, 2000; Howell and Shamir, 2005; Thoroughgood et al., 2012). Followers with low self-esteem are less likely to object to exploitation out of fear of rejection and disapproval. Weierter (1997) even goes as far as to argue that people with low self-esteem are more likely to identify with leaders who want to control and manipulate others, because those followers feel they deserve such treatment, thereby perpetuating a negative cycle of exploitation. It seems thus that persons with low self-esteem are susceptible to the influence of leaders with self-serving motivations.

Empirical support for this idea comes mostly from studies outside the leadership field. For instance, prior research on abusive behavior and workplace victimization points to low self-esteem, dependence, social isolation, and social incompetence as characteristics of potential victims (Harvey and Keashly, 2003; Zapf and Einarsen, 2003; Bowling and Beehr, 2006; Matthiesen and Einarsen, 2007). Moreover, those with low self-esteem are more likely to report problems related to being self-sacrificing and overly accommodating (Paz et al., 2017). Finally, research has shown that those with low self-esteem react to ego-threats with behavior that is more friendly, cautious and restrained, arguably because they are more focussed on belongingness needs and thus are focused on establishing a relationship with others, whether they respect them or not (Heatherton and Vohs, 2000; Vohs and Heatherton, 2001). Thus, those with low self-esteem may have less of a defense system against exploitation by those who are looking to further their own interests.

The compliant tendencies and the openness to exploitation of people with low self-esteem, however, may not be the only reason why those with low self-esteem may fall victim to the self-serving tendencies of leaders with psychopathy traits. In addition, subtle behavioral patterns and gestures of the low self-esteem followers might indicate that they offer little resistance in case of abuse (i.e., gestural hinting, see Grayson and Stein, 1981). It has been suggested that those with psychopathic traits are particularly capable of recognizing others’ vulnerability and have a willingness to exploit that. The infamous psychopathic serial killer Ted Bundy, for instance, boasted about his observational competencies by stating that he “could tell a victim by the way she walked down the street, the tilt of her head, the manner in which she carried herself, etc…” (as cited in Holmes and Holmes, 2009, p. 221). Several scholars indeed confirm that victims share certain characteristics that seem to predispose them for abuse and exploitation (Grayson and Stein, 1981; Richards et al., 1991; Gunns et al., 2002; Sakaguchi and Hasegawa, 2007). Other studies indicate that those with psychopathic traits are particularly likely to pick up on those characteristics (Book et al., 2013). For instance, Wheeler et al. (2009) found that psychopathic traits in a non-referred (and presumably not clinically psychopathic) sample increased the accuracy of perceptions of victim vulnerability. More recently, Demetrioff et al. (2017) found that individuals’ psychopathy scores were even positively associated with a heightened ability to identify sadness micro-expressions (note that low self-esteem often goes hand in hand with negative emotions) which further indicates their prowess in vulnerability assessment.

The idea of psychopaths being “social predators” (e.g., Hare, 2001; Book et al., 2007) hence seems to be justified. Boddy (2011) argues that such predatory behavior can be found in organizational contexts as well and conjectured that corporate psychopaths would mainly exploit those followers who are unlikely to defend themselves. The likelihood that leaders will engage in more self-serving behavior vis-a-vis followers that have low self-esteem might thus stem from followers’ own compliant tendencies, as well as from the psychopathic leader’s competencies in recognizing vulnerability and their willingness to take advantage of that. Notably, this resonates with Trait Activation theory (e.g., Christiansen and Tett, 2008), where it is argued that traits can be seen as latent propensities to behave in a certain way as a response to trait relevant cues (such as social cues). We posit that psychopathic traits in leaders carry the propensity to behave self-servingly as a response to cues that signal low self-esteem in followers. Given that expressing one’s traits is intrinsically satisfying, we expect that:

(1) *Hypothesis 2*: Leader psychopathy will be positively related to leader self-serving behavior to the extent that leaders are dealing with followers suffering from low self-esteem.

### Overview of the Present Research

We opted for a multiple-study, multiple-method approach so that comparable results between studies increase the confidence in our findings. In Study 1, a laboratory study with business leaders, we measured psychopathy and assigned all participants to a leader role. We then manipulated follower’s self-esteem, and asked the leader to perform a task in which they had the possibility to display self-serving behavior at the expense of the follower (using an ultimatum game). Study 2, was a multi-source field study (*N* = 124 unique leader–follower dyads) using questionnaires to assess leader psychopathy, follower self-esteem, and leader self-serving behavior as rated by the follower.

## Study 1

### Materials and Methods

#### Participants and Design

The study was conducted as an online survey of people in leadership positions from the United States, holding a job for at least 3 days a week. The 156 participants were randomly assigned to one of two conditions (Follower self-esteem: low vs. high), and participants’ psychopathy scores were added to the design as a continuous variable. Most of our participants worked in technology (17.9%), business and finance (17.3%), manufacturing (9.6%), education (8.3%), or human services (8.3%). Participants were predominantly male (57.7%), and their mean age was 36.33 (*SD* = 11.00). Most of them had a Bachelor degree or higher (76.3%), had been working on average for 16.07 years (*SD* = 10.46), and supervised on average 13.00 employees (*SD* = 34.86).

#### Procedure

Leaders were recruited using Amazon’s Mechanical Turk (Mturk). There are several scholars that advocate the use of Mturk data (Paolacci et al., 2010; Buhrmester et al., 2011; Mason and Suri, 2012), also specifically for organizational research (e.g., Rietzschel et al., 2017), as well as studies that have actually used Mturk for collecting leader data. Van Houwelingen et al. (2017), for example, used Mturk for sampling leaders and found results comparable to other samples they used in the same paper^1^. Participants were informed that the research would take approximately 15 min to complete, that the data collected with this study would be treated confidentially, and that they would receive $1.30 in return for their participation. Participants also learned that they had the opportunity to earn a bonus payment (based on task performance).

The study consisted of two parts. In the first part, participants provided their informed consent via the program software and filled out several questionnaires - including our psychopathy measure. In the second part, participants played an ultimatum game with a fictitious other Mturk worker, with whom they were allegedly randomly paired up. Participants were told that, based on a comparison of their own and the other person’s answers on the questions in the first part, one of them would be assigned the role of leader and the other one the role of subordinate. In reality, all participants were assigned the leader role (there were no subordinates). Next, participants were presented with the instructions for the task – an ultimatum game – in which they were to divide a bonus payment between themselves and their subordinate (the fictitious other Mturk Worker). Participants then performed the task, answered some (demographical and manipulation check) questions, were debriefed, thanked, and paid (a base pay of $1.30 and a bonus [up to $0.60]). The experimental procedure was approved by the Ethics Committee of Psychology of the University of Groningen.

#### The Experimental Task: An Ultimatum Game

In a (symmetrical) ultimatum game people are asked to divide money or other rewards between themselves and another person (Handgraaf et al., 2003). The game usually has two players: an allocator and a recipient. The allocator is asked to divide the money, and the recipient has the chance to either accept or reject the offer made by the allocator. If the recipient accepts the offer, the money will be divided based on the proposal made by the allocator. If the recipient rejects the offer, both get nothing. In a symmetrical ultimatum game, both the allocator and the recipient know how much money can be divided (symmetric information; e.g., van Dijk and Vermunt, 2000). As keeping more money for oneself automatically results in less money for the other, the game has been used in previous research to assess self-serving behavior that comes at the expense of another person (van Dijk and Vermunt, 2000; Sanders et al., 2016). In our experiment, all participants had the allocator role and had to decide on the distribution of a bonus payment of 60 dollar cents between themselves and their (fictitious) subordinate. The allocators did not know that there were no actual recipients. The ultimatum game in this case is strictly speaking not symmetrical, since there are no real recipients involved.

#### Self-Esteem Manipulation

Before playing the ultimatum game, participants received some information about their subordinate. Specifically, participants were presented with a table displaying their subordinate’s alleged scores on the first five items of the Rosenberg (1965) self-esteem scale, which we used to manipulate the subordinate’s level of self-esteem. In the low self-esteem condition, participants could see that the recipient scored a 1 or a 2 on a 7-point scale on items such as: “On the whole I am satisfied with myself.” In the high self-esteem condition, participants could see that the recipient scored a 6 or a 7 on these items.

#### Measures

##### Psychopathy

Leaders’ psychopathic traits were assessed with Levenson’s Self-Report Primary Psychopathy Scale (LSRPA; Levenson et al., 1995). This 16-item scale for the assessment of primary psychopathy includes items such as: “I enjoy manipulating other people’s feelings” and “For me, what is right is whatever I can get away with.” Leaders indicated their agreement with the statements using a 4-point Likert scale (1 = *disagree strongly*, 4 = *agree strongly*). In a recent study, Tsang et al. (2018, p. 316) found that the items of the Levenson scale “assessing primary psychopathy are better at differentiating between individuals with varying levels of psychopathic traits than items measuring secondary psychopathy features.” This study also found confirmatory support for the primary, but not the secondary psychopathy scale. The reliability of the primary psychopathy scale in the present study was very good with Cronbach’s alpha = 0.93 (*M* = 1.86; *SD* = 0.59).

##### Manipulation check

To assess the effectiveness of the self-esteem manipulation, we used the final five items of Rosenberg’s (1965) self-esteem scale. Participants were for instance asked to what extent the other person “…has a positive attitude toward him/herself,” “…is inclined to feel that he/she is failure” (R). Participants’ responses were assessed using a 5-point Likert scale (1 = *not at all*, 5 = *extremely*). Cronbach’s alpha of the scale = 0.96 (*M* = 2.99; *SD* = 1.46).

##### Leader self-serving behavior

The number of cents leaders allotted to themselves (at the expense of their subordinate) in the ultimatum game comprised our behavioral measure of leaders’ self-serving behavior (*M* = 33.43; *SD* = 6.71).

##### Controls

We controlled for supervisor age (Barlett and Barlett, 2015), and gender (Webster and Jonason, 2013; coded 1 = *male*; 2 = *female*), because previous research found these variables to be related to psychopathy (cf. Jonason and Webster, 2010; Wisse and Sleebos, 2016).

### Results

#### Manipulation Check

An independent samples *t*-test showed that participants in the high self-esteem condition (*M* = 4.31, *SD* = 0.61) perceived their subordinate to have higher self-esteem than those in the low self-esteem condition (*M* = 1.70, *SD* = 0.66), *t*(154) = -25.57, *p* < 0.001 (mean difference = -2.61, 95% CI = [-2.81, -2.41]). These results demonstrate that the manipulation worked as intended.

#### Leader Self-Serving Behavior

**FIGURE 1:**
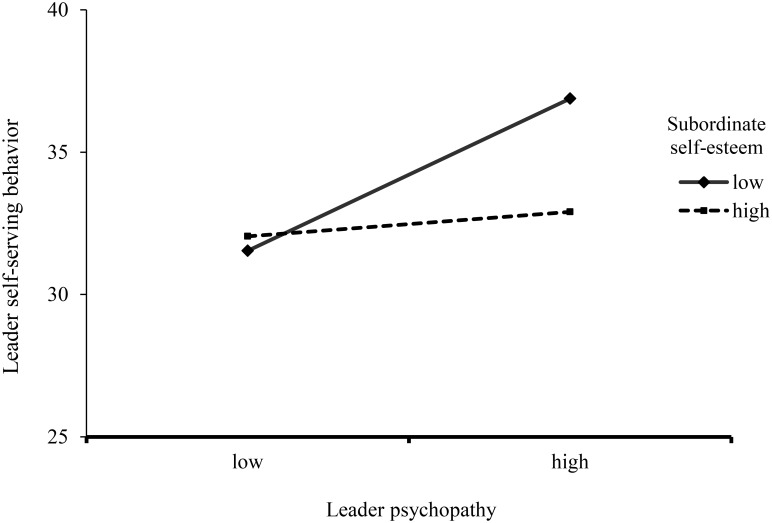
Self-serving behavior as indicated by cents allotted to self as a function of leader psychopathy and manipulated subordinate self-esteem.

We predicted that leader psychopathy and subordinate self-esteem would interact in such a way that particularly when subordinate self-esteem is low, leader psychopathy would be related to self-serving behavior. To test this hypothesized moderation, we relied on a procedure suggested by Hayes (2013; model 1; see **Table 1**). We controlled for supervisor age and gender. We found a main effect of supervisor psychopathy, showing that supervisors were more self-serving when they scored higher on psychopathy. In addition, and in line with our hypothesis, we found that the interaction term of supervisor psychopathy and employee self-esteem significantly predicted the amount of money that the supervisor took for him/herself. We tested the conditional direct effects of supervisor psychopathy on the dependent variable (self-serving behavior) at different levels of employee self-esteem. Bootstrapping (5,000 samples) confirmed that the direct effect of supervisor psychopathy on self-serving behavior was significant for employees with low self-esteem (*b* = 4.49, 95% CI = [2.08, 6.90]; 1 *SD* below the mean), but not for employees with high self-esteem (*b* = 0.73, 95% CI = [-2.09, 3.55]; 1 *SD* above the mean) (**Figure 1**).

**Table 1:** Regression results for the (conditional) effects of Study 1.

Predictor		Moderator model		
		
		(DV = self-serving behavior)		
		b^a^	*SE*	*t*(156)
Constant		26.14	3.95	6.62^∗∗^
Gender		0.23	1.09	0.21
Age		- 0.02	0.05	- 0.31
Supervisor psychopathy		4.49	1.22	3.68^∗∗^
Employee self-esteem		5.24	3.43	1.53
Psychopathy × Self-esteem		- 3.76	1.77	- 2.12^∗^

	**Conditional effects**			
	** at values of the moderator**			
	
	**Effect**	**Boot SE**	**BootLLCI**	**BootULCI**

Low self-esteem	4.49	1.22	2.08	6.90
High self-esteem	0.73	1.43	-2.09	3.55


*^*a*^Unstandardized regression coefficients; ^∗^*p* < 0.05, ^∗∗^*p* < 0.01.*

## Study 2

### Materials and Methods

#### Respondents

We approached 300 dyads of Dutch subordinates and their direct supervisors. After initial screening (removing respondents who did not fill out all psychopathy, self-serving behavior or self-esteem questions), we had a dataset of 124 dyads (response rate 41.33%). A total of 42.7% of the subordinates and 65.3% of the supervisors indicated to be male. Subordinates’ mean age was 31.20 (*SD* = 11.52) and supervisors’ mean age was 40.48 (*SD* = 11.07). A total of 65.0% of the supervisors and 30.6% of the subordinates indicated having worked more than 5 years in their current position. Most supervisors and subordinates worked more than 25 h a week (92.7% and 53.2%, respectively). The majority of our respondents worked in commercially oriented (service) organizations (e.g., shops, financial institutions, health care organizations, etc.; 73.3%).

#### Procedure

Data were collected as part of a study on the role of personality in the workplace. Research assistants used their own work environment, their personal network and that of acquaintances to get in contact with employees and supervisors. In addition, they actively visited business and shopping centers. Potential participants were approached via email, through phone calls, or face-to-face contact. We stressed the fact that participation was voluntary and that data would be treated confidentially. If subordinates and their supervisors were interested in participating, they were asked to fill out the paper-and-pencil questionnaires without consulting their colleagues, subordinates or supervisor, and to return the questionnaires in the enclosed envelope. This envelope was then picked up by the research assistant or returned by mail. Because people often filled out the questionnaires during work hours, we kept the survey short and to the point. Respondents also had the option to fill in the questionnaire during their free time (e.g., during lunch breaks or at home). Participants gave their informed consent, and the study was approved by the Ethics committee of the University of Groningen.

#### Measures

The following measures were used in this study:

##### Psychopathy

Leaders’ primary psychopathy was again assessed by asking supervisors to fill out LSRPA (Levenson et al., 1995; 1 = *disagree strongly*, 4 = *agree strongly*).

##### Self-esteem

To measure the self-esteem of the follower, we asked subordinates to fill out the 10 items of the Rosenberg Self-Esteem Scale (Rosenberg, 1965), using a 5-point Likert scale (1 = *completely disagree*, 5 = *completely agree*).

##### Leader self-serving behavior

Perceptions of the degree to which leaders demonstrated self-serving behavior were assessed using the scale developed by Rus et al. (2010). Because, we asked subordinates to assess their leader’s behavior, we removed one item from the original 8-item scale as subordinates generally do not have access to that information (“My supervisor negotiated a bonus for him/herself that was substantially higher than the bonus we receive”). The scale includes items such as “My supervisor has used his/her leadership position to obtain benefits for him/herself,” and “Instead of giving credit to me or my colleagues for jobs requiring a lot of time and effort, my supervisor took the credit him/herself”). Subordinates rated their leaders’ self-serving behavior using a 4-point Likert scale (1 = *never*, 4 = *often*).

##### Controls

We again controlled for supervisor age and gender (1 = *male*; 2 = *female*). Additionally, we controlled for length of collaboration and frequency of contact (as indicated by the subordinate) because previous research suggests that others’ perceptions of people scoring high on Dark Triad traits may change once they get to know them better (cf. Campbell and Campbell, 2009). Length of collaboration was assessed using five categories which were coded 1 (less than 6 months) to 5 (5 years or longer). Frequency of contact was assessed using a 5-point scale (1 = *sporadic*; 5 = *very often*)^2^.

### Results

**Table 2** shows means, standard deviations, zero-order Pearson correlations, and Cronbach’s alphas for the study variables^3^. Cronbach’s alpha’s were all sufficiently high. Note that, we found a significant positive correlation between leader psychopathy and perceptions of leader self-serving behavior (*r* = 0.24, *p* < 0.01)

**Table 2:** Descriptives and correlations for Study 2 variables.

Variable	*M*	*SD*	1	2	3	4	5	6	7
**Supervisor rated**								
1. Gender	–	–	–						
2. Age	40.48	11.07	-0.19*						
3. Psychopathy	2.18	0.37	-0.13	-0.09	(0.79)				
**Subordinate rated**								
4. Length of collaboration	3.23	1.43	-0.01	0.24**	-0.00	–			
5. Frequency of contact	3.83	0.99	0.04	-0.03	-0.10	0.10	–		
6. Self-esteem	4.12	0.47	-0.02	-0.03	0.03	0.16	-0.04	(0.79)	
7. Self-serving behavior	1.41	0.51	-0.05	0.12	0.24**	0.19*	-0.18*	-0.16	(0.88)


**N* = 124; ^∗^*p* < 0.05; ^∗∗^*p* < 0.01 (two-tailed); Cronbach’s alpha between brackets.*

#### Leader Self-Serving Behavior

**FIGURE 2:**
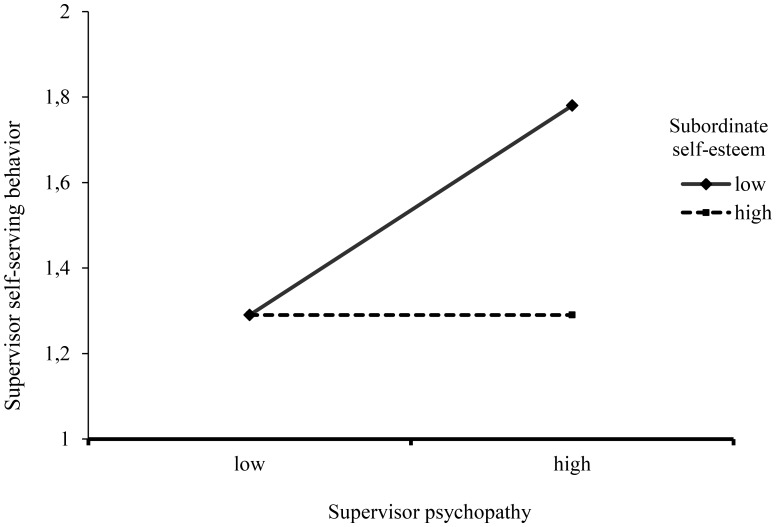
Subordinate rated self-serving behavior of supervisor as a function of supervisor psychopathy and subordinate self-esteem.

We predicted that leader psychopathy would be more strongly related to (perceived) leader self-serving behavior to the extent that subordinate self-esteem is low. To test this hypothesized moderation, we again relied on Hayes (2013; model 1; see **Table 3**). We controlled for supervisor age, gender and length of collaboration and frequency of contact. We found a main effect of supervisor psychopathy, showing that supervisors were rated as more self-serving when they scored higher on psychopathy. We also found a main effect of employee self-esteem, showing that employees perceived more self-serving behavior when they had lower self-esteem. In addition, and in line with our hypothesis, we found that the interaction term of supervisor psychopathy and employee self-esteem significantly predicted (perceptions of) leader self-serving behavior. We tested the conditional direct effects supervisor psychopathy on the dependent variable (self-serving behavior) at different levels of employee self-esteem. Bootstrapping (5,000 samples) confirmed that the direct effect of supervisor psychopathy on (perceptions of) leader self-serving behavior was significant for employees with low self-esteem (*b* = 0.67, 95% CI = [0.33, 1.02]; 1 *SD* below the mean), but not for employees with high self-esteem (*b* = 0.00, 95% CI = [-0.32, 0.33]; 1 *SD* above the mean) (also see **Figure 2**).

**Table 3:** Regression results for the (conditional) effects of Study 2.

Predictor		DV = self-serving behavior		
	
		b^a^	*SE*	*t*(123)
Constant		1.51	0.28	5.33^∗∗^
Gender		-0.05	0.09	-0.50
Age		0.00	0.00	0.94
Collaboration length		0.07	0.03	2.08^∗^
Frequency of contact		-0.10	0.04	-2.42^∗^
Supervisor psychopathy		0.34	0.12	2.94^∗∗^
Employee self-esteem		-0.26	0.09	-2.89^∗∗^
Psychopathy × Self-esteem		-0.71	0.26	-2.68^∗∗^

	**Conditional effects**			
	** at values of the moderator**			
	
	**Effect**	**Boot SE**	**BootLLCI**	**BootULCI**

Low self-esteem	0.67	0.17	0.33	1.02
High self-esteem	0.00	0.17	-0.32	0.33


*^*a*^Unstandardized regression coefficients; ^∗^*p* < 0.05, ^∗∗^*p* < 0.01.*

## Discussion

This study focused on leader psychopathic personality traits, follower self-esteem and leader self-serving behavior. Across two studies, one experimental study and one field study, we found that leader psychopathy positively predicts (perceived) leader self-serving behavior. More importantly, we found that follower self-esteem moderated the relationship between leader psychopathy and leader self-serving behavior. Only when followers had low self-esteem, we found that leader psychopathy and leader self-serving behavior were positively related. The results support and extend previous studies in several ways.

First, the study adds to the growing list of potential consequences of employing people with psychopathic tendencies by showing that leader psychopathy is associated with self-serving behavior. Given the adverse effects that leader self-serving behavior may have on outcomes for subordinates and for organizations (Carmeli and Sheaffer, 2009; Peterson et al., 2012) it is crucial to understand its determinants. Notably, the occurrence of self-interested behavior without heeding to the needs of others may prove particularly detrimental when larger resources are at stake (Wisse and Rus, 2012). This renders leader behavior particularly important, because leaders tend to have more control over resources than rank and file employees.

Second, the study indicates that followers can have an effect on the extent to which leaders’ psychopathic traits will be reflected in their behavior. This finding thus confirms the notion that destructive organizational outcomes are not exclusively the result of destructive leaders, but are also products of ‘susceptible followers’ (Padilla et al., 2007; Thoroughgood et al., 2012). Thoroughgood et al. (2012) distinguished between two classes of susceptible followers: the conformers and the colluders. While conformers are prone to obedience, colluders actively contribute to the leaders’ mission. In our study, we have considered the influence of low self-esteem; a characteristic that is likely to make a follower belong to the conformer category. These followers are considered vulnerable to leaders wishing to exploit them, arguably out of a fear of confrontation that creates a weakness to social pressures, or out of a need to gain the approval of someone who is able to provide clarity, direction, and increased self-esteem. Perhaps future research could focus on if the relationship between leader psychopathy and self-serving behavior is also strengthened by the presence of ‘authoritarian’ followers. Authoritarians also belong to the conformer category, but these follower possess rigid, hierarchical attitudes that prescribe leaders’ legitimate right to exert (Altemeyer, 1998), and those with psychopathic traits may be inclined to make use of that to their own advantage. Moreover, future research may want to investigate the role of colluders in more detail. Anecdotal evidence suggests that psychopaths may sometimes work through or with their ‘henchmen’ (Babiak and Hare, 2006; Langbert, 2010) to accomplish their self-serving goals, but more research on the matter is needed.

In addition, our study suggests that those with higher levels of psychopathy differentiate between the one person and the other in terms of victim selection. That is, leaders with psychopathic traits victimized followers with low self-esteem more than those with high self-esteem. Interestingly, a recent study (Black et al., 2014) argued that psychopathic personalities may not be so ‘picky’ when choosing a victim. This study examined the relation between the Dark Triad (psychopathy, Machiavellianism, and narcissism) and strategies used in the assessment of personality and emotional states related to vulnerability in others. Their results indicated that dark personalities engaged in a relatively superficial interpersonal analysis and generally perceived all targets as weak and vulnerable. The authors proposed that instead of being keen “readers” of others, dark personalities, including psychopathic ones, may rely on their ability to draw in vulnerable victims (for instance based on their charisma or good looks) or adopt a “quantity over quality” strategy to find targets and then use manipulation tactics to exploit them. Our studies’ results are more in line with other findings that suggest that psychopaths do differentiate between targets (Wheeler et al., 2009; Book et al., 2013; Demetrioff et al., 2017). Perhaps future research could look at potential moderators in order to explain when psychopaths make a distinction between potential targets and when they do not.

We would like to draw attention to a couple more issues that could fruitfully be addressed in future research. For instance, we mentioned that both a lack of self-esteem as well as the surplus in psychopathy may set in motion certain process that may explain why those with low self-esteem may fall prey to the exploitative tendencies of those scoring high on psychopathy. Indeed, we argued that low self-esteem may engender compliant behavior and vulnerability on the part of the follower, and that psychopathy may come with a knack for recognizing vulnerability and the willingness to misuse that on the part of the leader. Although we indeed find that leader psychopathy and follower self-esteem interact to explain leader self-serving behavior, our results are mute when it comes to the underlying process. Of course, in the experiment the role of the follower was manipulated and there was no actual follower present, but in the field study the follower did exist and his/her behavior may have set exploitative processes in motion. Therefore, it remains unclear to what extent various processes are set in motion by leader psychopathy and follower self-esteem that may explain their combined effect. Future studies could explore the potential mediating roles of for instance compliance, a focus on vulnerability cues, etc. Moreover, it may be worthwhile to examine the implications of psychopathy for self-serving behavior over time using longitudinal designs (Smith and Lilienfeld, 2013). Psychopathy may be adaptive for first impressions, but over time co-workers and employers may begin to grow weary of psychopathic individuals and associated self-serving tendencies. It would be valuable to get more insight into to the long term development of leader exploitive behavior of followers.

### Strengths and Limitations

As with every study, the present study has its strengths and limitations. One strength is that by conducting both an experiment and a field study, we adopted a multiple-study, multiple-method approach in which the strengths of one method may compensate for any weaknesses in the others (Eid and Diener, 2006). For instance, the multiple method approach allowed us to assess leader self-serving behavior in different ways. In the field study, we asked followers to indicate the extent to which their leader displayed self-serving behavior. With this approach, we followed contemporary practices in leadership research and recommendations for research on consequences of Dark Triad personalities in the workplace (Smith and Lilienfeld, 2013). The use of subordinate perception data has the advantage of not having to rely on leaders own perceptions of their self-serving behavior (and thus of avoiding self-serving bias on the part of the leader). However, one potential drawback is that perceptions of observers and reality may differ as well (Hansbrough et al., 2015). Indeed, one may argue that those with low self-esteem are plainly more aware of or sensitive to negative behaviors of others, or that that their perception of negative behaviors of others is more negative than the perception of those with high self-esteem. Our experimental study shows, however, that these arguments cannot satisfactorily explain our findings. After all, in the experiment, we assessed self-serving behavior using a behavioral measure instead of a perception measure. In this study, we also found that leader psychopathy is only related to self-serving tendencies in the actual division of monetary rewards when the follower has low self-esteem.

Other compensatory advantages of the present paper’s multi-method approach are, for instance, that even though we took special care to achieve a high degree of experimental realism, the experiment could still be criticized for its artificial character. That is, findings generated in an experimental environment provide no evidence that the same relationships actually exist outside the laboratory (Goodwin et al., 2000). Moreover, although an ultimatum game may be perceived as a simple form of leader–follower exchange (Price and Van Vugt, 2014), and ultimatum games have been used in previous research to assess self-serving behavior that comes at the expense of another person (e.g., van Dijk and Vermunt, 2000; Sanders et al., 2016), one might question the ecological validity of such a game. Our second study may alleviate these concerns as it shows that these relationships may indeed be observed in the field.

Another limitation of the present study is that we only examined primary psychopathy, not secondary psychopathy. Because primary psychopathy, contrary to secondary psychopathy, does not seem to hinder individuals from functioning reasonably well in society, and also appears to capture the core of the psychopathy concept as defined by Cleckley (1964; e.g., Murphy and Vess, 2003), we focused on primary psychopathy only. Future studies might, however, also examine secondary psychopathy, in order to examine the effect of both elements in the Dual Process model of psychopathy on leader self-serving behavior, or, additionally, all three Dark Triad traits.

### Practical Implications

Several scholars have cautioned against studying the role of victim personality and suggested that one has to be careful with respect to these issues, in order to avoid being accused of “blaming the victim” (Zapf and Einarsen, 2003). Of course, in studying victim personality, it is not our intention to hold victims responsible for their exploitation, neither do we suggest that others should do so. However, we hope our study makes clear that there are legitimate reasons to examine follower personality and the role it may play in self-serving leadership. Developing effective intervention techniques in order to prevent self-serving behavior by leaders depends upon a comprehensive understanding of the phenomenon. If follower personality and associated behaviors and needs (non-verbal behavior, compliance, need to belong) trigger self-serving behavior in leaders, followers are better off if they are aware of it so they can address it. Moreover, even if follower personality plays role in self-serving behavior of leaders, that does not mean that organizations and employers are cleared of a responsibility in the prevention and termination of such behaviors in the workplace. On the one hand organizations may assist targets in addressing or dealing with the issue, and on the other hand they may want to hold self-serving leaders accountable for their actions. Indeed, it has been suggested that accountability, a lack of ambiguity, and a clear set of values and norms may mitigate the negative impact of psychopaths in the workplace (Cohen, 2016). Moreover, the results of this study give further credence to the idea that organizations may want to be cautious with promoting those with dark personalities into positions of leadership (Wisse and Sleebos, 2016). Notably, to prevent those with higher levels of psychopathic traits from being promoted to or hired into leadership positions more research on how to screen for psychopathic traits is needed (Stevens et al., 2012; Spain et al., 2014).

All in all, given the negative consequences of leader self-serving behavior for subordinates as well as for the organization at large, more insight into the conditions that prompt supervisors to engage in such behavior is essential. We found that follower self-esteem and leader psychopathy jointly determined leader self-serving behavior, as such that leader psychopathy predict leader self-serving behavior to the extent to followers had low self-esteem. We hope that our study stimulates research that employs an interactionist perspective (integrating both leader and follower characteristics) on the influence of corporate psychopaths at work. This may further insight into how organizations can protect themselves against the destructive influences of supervisors with psychopathic traits.

## Author Contributions

All authors provided substantial contributions to the conception or design of the work; were responsible for drafting the work or revising it critically for important intellectual content; approved the final version of this manuscript; and agreed to be accountable for all aspects of the work.

## Conflict of Interest Statement

The authors declare that the research was conducted in the absence of any commercial or financial relationships that could be construed as a potential conflict of interest. The reviewer IK and handling Editor declared their shared affiliation at the time of review.

## Footnotes

1 In line with recommendation for studies using Mturk samples, we deleted participants who indicated, we should not use their data or had absolute studentized residuals larger than 3 (Cheung et al., 2017).

2 The present study was part of a large-scale study that also included scales for the other two Dark Triad traits: Machiavellianism (a shortened version of the MACH-IV; Belschak et al., 2015) and narcissism (the NPI-16; Ames et al., 2006). Because, we focus on psychopathy here, we did not use these scales. These scales were also not available in Study 1.

3 The structure of the items assessing self-esteem, leader self-serving behavior, and psychopathy was examined by means of EFA. The EFA produced three oblique factors that very closely resembled the three *a priori* scales. Congruencies (Tucker’s phi; Tucker, 1951) between the three EFA factors and the three *a priori* scales were subsequently computed in Matlab. The congruencies, after orthogonal Procrustes rotation (Kiers and Groenen, 1996), were: self-esteem φ = 0.95, leader self-serving behavior φ = 0.91, and psychopathy φ = 0.90. These results support the expected structure of the data (e.g., Lorenzo-Seva and Ten Berge, 2006).
